# Comparative Analysis of Microbial Consortiums and Nanoparticles for Rehabilitating Petroleum Waste Contaminated Soils

**DOI:** 10.3390/molecules27061945

**Published:** 2022-03-17

**Authors:** Shehla Sattar, Samina Siddiqui, Asim Shahzad, Asghari Bano, Muhammad Naeem, Rahib Hussain, Naeem Khan, Basit Latief Jan, Humaira Yasmin

**Affiliations:** 1 National Centre of Excellence in Geology, University of Peshawar, Peshawar 25130, Pakistan; samina.siddiqui@gmail.com (S.S.); rahib.cees@pu.edu.pk (R.H.); 2 Department of Environmental Sciences, University of Swabi, Swabi 23561, Pakistan; 3 Department of Botany, Mohi-ud-Din Islamic University, Nerian Sharif 12080, Pakistan; agron12@yahoo.com; 4 College of Geography and Environment, Henan University, Jinming Ave, Kaifeng 475004, China; 5 Department of Bio-Sciences, Quaid Avenue University of Wah, Wah 47000, Pakistan; asghari.bano@uow.edu.pk; 6 Department of Biotechnology, Mohi-ud-Din Islamic University, Nerian Sharif 12080, Pakistan; naeembbt@gmail.com; 7 Institute of Crop Germplasm Resources, Shandong Academy of Agricultural Sciences, Jinan 250100, China; 8 College of Earth and Environmental Sciences, University of the Punjab, Lahore 54590, Pakistan; 9 Department of Agronomy, Institute of Food and Agricultural Sciences, University of Florida, Gainesville, FL 32611, USA; naeemkhan@ufl.edu; 10 Department of Clinical Pharmacy, College of Pharmacy, King Saud University, Riyadh 11451, Saudi Arabia; bjan@ksu.edu.sa; 11 Department of Biosciences, COMSATS University Islamabad (CUI), Islamabad 45550, Pakistan

**Keywords:** nano-bioremediation, consortium, nanoparticles, petroleum degradation, total petroleum hydrocarbons, chemical fertilizer, soil contamination

## Abstract

Nano-bioremediation application is an ecologically and environmentally friendly technique to overcome the catastrophic situation in soil because of petroleum waste contamination. We evaluated the efficiency of oil-degrading bacterial consortium and silver nanoparticles (AgNPs) with or without fertilizer to remediate soils collected from petroleum waste contaminated oil fields. Physicochemical characteristics of control soil and petroleum contaminated soils were assessed. Four oil-degrading strains, namely *Bacillus pumilus* (KY010576), *Exiguobacteriaum aurantiacum* (KY010578), *Lysinibacillus fusiformis* (KY010586), and *Pseudomonas putida* (KX580766), were selected based on their in vitrohydrocarbon-degrading efficiency. In a lab experiment, contaminated soils were treated alone and with combined amendments of the bacterial consortium, AgNPs, and fertilizers (ammonium nitrate and diammonium phosphate). We detected the degradation rate of total petroleum hydrocarbons (TPHs) of the soil samples with GC-FID at different intervals of the incubation period (0, 5, 20, 60, 240 days). The bacterial population (CFU/g) was also monitored during the entire period of incubation. The results showed that 70% more TPH was degraded with a consortium with their sole application in 20 days of incubation. There was a positive correlation between TPH degradation and the 100-fold increase in bacterial population in contaminated soils. This study revealed that bacterial consortiums alone showed the maximum increase in the degradation of TPHs at 20 days. The application of nanoparticles and fertilizer has non-significant effects on the consortium degradation potential. Moreover, fertilizer alone or in combination with AgNPs and consortium slows the rate of degradation of TPHs over a short period. Still, it subsequently accelerates the rate of degradation of TPHs, and a negligible amount remains at the end of the incubation period.

## 1. Introduction

Several tonnes of petroleum wastes are produced during oil exploration. Petroleum waste is a mixture of water, emulsion, solids, and liquid crude oil [1]. When released to the environment, this waste may cause severe threats to the agricultural–geographical (agri–geo) environmental ecosystem of the surrounding areas [2]. Soil, water, and air are integral parts of the agri–geo environmental ecosystem and are at risk because of the hazardous and carcinogenic nature of the presence of some organic compounds in petroleum waste [3]. These hazardous and carcinogenic compounds, when sporadically spilling over soil from petroleum waste pits, may cause soil contamination. Thus, such soil becomes infertile and unable to support flora and fauna [4,5,6,7].

Bioremediation, bioaugmentation, and composting (biological methods) are preferred over the available chemical and physical methods because they are cost-effective, environmentally friendly, and socio-economically acceptable to communities. Biological methods such as bioremediation have some limitations, including the selection of appropriate biodegrades, available nutrients, optimum viscosity, and suitable concentration of petroleum waste [8]. For these reasons, further improvements in bioremediation with plants, surfactants, and nano-technology are gaining attention among researchers.

Limitations in bioremediation revealed the contention that the complete remediation of petroleum waste contaminated soils requires some extra accelerator having nontoxic characteristics and a large surface area to accelerate the bioremediation of petroleum waste completely [9]. Nanoparticles, having high adsorption capacity, minimum reactivity, and releasing nontoxic ions, may increase their suitability to remediate petroleum waste contaminated soils [10]. The removal of both organic and inorganic contaminants by using nanoparticles is focused on today due to their effectiveness and efficacy [11,12]. Nanoparticles, such as silver and gold, provide a large surface area for microbes to degrade the contaminants rapidly [13,14]. Nanoparticles (because of greater surface area) can adsorb heavy metals, thus enhancing their assimilation. Saber and colleagues [15] applied iron oxide NPs to remove crude oil from water. Because of their environmental feasibility, ecologically sound behavior, greater adsorption capacity, minimum reactivity, the release of nontoxic ions, and availability of large surface area for microbes, nanoparticles were successfully applied at a commercial scale to remediate the industrial effluents [16].

Several studies explored the role of microbes or nanoparticles in the degradation of petroleum waste [17,18]. However, the synergistic role of microbes with AgNPs in terrestrial environments has not been properly understood. Therefore, the effect of AgNPs individually, as well as in combination with consortium and fertilizer, needs proper investigation. To the best of our knowledge, the role of microbes with AgNPs in combination with consortium and fertilizer in the degradation of petroleum waste contaminated soils has never been assessed. Moreover, it was evaluated by using strains found in oil-contaminated sites, namely *Bacillus pumilus* KY010576, *Pseudomonas putida* (KX580766), *Exiguobacterium aurantiacum* (KY010578), and *Lysinibacillus fusiformis* (KY010586), which have never been tested before for their oil degradation abilities. The application of consortium with *Exiguobacteriaum aurantiacum*(KY010578) and *Lysinibacillus fusiformis* had not been tested previously to evaluate the fate and effect of total hydrocarbon degradation in petroleum waste contaminated soil over time. The present study aimed to investigate the synergistic use of a microbial consortium with AgNPs to degrade petroleum waste contaminated soils.

## 2. Material and Methods

### 2.1. Survey and Sample Collection

Petroleum waste pits were found within the vicinity of Chanda and Mela oil fields in Kohat Plateau. A systematic soil sampling technique was adopted for control and petroleum waste contaminated soils. A plot of ten to 20 m^2^ surrounding the petroleum waste pit was selected. Control soil samples were collected from the surrounding area near the oil field. Triplicate petroleum waste contaminated soil samples were collected from the exterior of petroleum waste pit (L × W × D) within the vicinity of oil fields and were placed in zipper bags and brought to the Petroleum Geochemistry Laboratory of National Centre of Excellence of Geology, the University of Peshawar, Pakistan, for further analysis.

### 2.2. Physicochemical Characteristics of Control Soil and Petroleum Contaminated Soils

Control soil and petroleum waste contaminated soils were analyzed for soil pH, EC (µS/cm), soil texture, organic matter, total nitrogen, and total phosphorus by Spark [19]. For total bacterial population/viable counts of bacteria in all samples, serial dilutions were made by removing 1 g of soil/petroleum waste from the bulk sample, which was then diluted with 9 mL of sterile water according to the method described by Song and Bartha [20].

### 2.3. Selection of Microbes for Consortium Preparation

Three microbes were isolated from oil fields of the Potwar Plateau but applied to Chanda oil fields for the degradation experiment. These were *Bacillus pumilus*, *Exiguobacterium aurantiacum*, and *Lysinibacillus fusiformis*, whereas *Pseudomonas putida* were collected from the Department of Biosciences, University of Wah. *Pseudomonas putida* was previously isolated by Naseem and Bano [21].

To select microbes for the preparation of bacterial consortium, the hydrocarbon-degrading capacity of isolated strains was determined. The hydrocarbon-degrading efficiency of isolated strains was screened on nutrient agar plates by using petroleum waste hydrocarbons as the sole source of energy. The bacterial strains capable of degrading petroleum hydrocarbons were selected for the preparation of the consortium.

### 2.4. Gene Sequence-Based Identification (16S rRNA) of Bacteria

The strains were identified by molecular techniques based on 16S rRNA gene sequencing and BLAST homology by using a method proposed by Webb and Maas [22].

### 2.5. Phylogenetic Relationship of Bacteria

To determine the phylogenetic history of the isolated strains, the neighbor-joining method of Saitou and Nei [23] was followed. The phylogenic tree/dendrogram of isolated strains were developed using molecular evolutionary genetics analysis by Molecular Evolutionary Genetics Analysis (MEGA 6) software [24].

### 2.6. Consortium Preparation

The bacterial strains used in the consortium (prepared from *Bacillus pumilus* KY010576, *Exiguobacteriaum aurantiacum* KY010578, *Lysinibacillus fusiformis* KY010586, and *Pseudomonas putida* (KX580766))were isolated from surface and subsurface horizons of oil fields of Potwar Plateau in Pakistan and identified by 16S rRNA gene sequencing.

Around 13 g of nutrient broth agar was placed in a culture autoclave bottle and diluted with 1 L of distilled water. The suspension was sterilized at 121 ± °C for 45 min. The sterilized nutrient broth (100 mL) was poured over the flask (250 mL) following the method described by Patowary and colleagues [25]. The individual fresh colony (24 hold colony) from each bacterial strain (used to prepare consortium) was picked and inoculated in the flask with broth. The flask was closed with a cotton plug to avoid contamination during incubation. All flasks were incubated at 150 rpm for 7 days using a shaking incubator (Shin Saeng IRMECO-Kangwon, Paju, Korea). The growth of bacterial strains was monitored every 24 h. Thereafter, the nutrient broth media with a fresh single bacterial colony (selected from the isolated strains) were centrifuged at 5000 rpm for 10 min. The supernatant was discarded, and pellets were obtained.

Each pellet was re-suspended individually in autoclaved distilled water and the optical density (O.D) was adjusted to 1 at 660 nm with a UV-VIS spectrophotometer (Shimadzu). The consortium was prepared by mixing pure cultures of bacterial strains in an equal volume of an individual strain having O.D 1 at 660 nm and bacterial density (106 cells/mL). The total viable count or colony-forming units (CFUs/g) of oven-dry soil was determined through Equation (1).

(1) $$\frac{\mathrm{CFU}}{g\;\left(\mathrm{soil}\right)}=\frac{\mathrm{Number}\;\mathrm{of}\;\mathrm{colonies}}{\mathrm{Volume}\;\mathrm{of}\;\mathrm{inoculum}}\times \mathrm{Dilution}\;\mathrm{factor}$$

### 2.7. Experimental Design

To examine the role of the bacterial consortium, AgNPs, and fertilizers in the degradation of total petroleum hydrocarbons, a lab experiment was conducted. The petroleum waste contaminated soils were treated with a consortium, AgNPs, and fertilizers alone and/or in combination (T1–T7), as shown in Table 1. The treated (T1–T7) and untreated/control (T0) soil samples were further analyzed by GC-FID at different intervals of the incubation period (5, 20, 60, and 240 days) to investigate the rate of degradation of hydrocarbons with respect to time.

### 2.8. GC-FID Analysis

To extract crude oil from petroleum waste samples, the Soxhlet extraction procedure was adopted. Approximately10 g from each untreated (T0) and treated (T1–T7) soil sub-sample were removed and placed in the known weight of cellulose thimble. Thereafter, the soil with cellulose thimble was placed over the condenser. The solvents were added at the ratio of 4:1:1 of dichloromethane: n-hexane: methanol in the round bottom flask (250 mL). Each flask with solvent was placed over the heater and heated overnight. To ensure complete extraction, each sample was extracted for a further 2 h before being removed. Thereafter, the mixture was removed from the round bottom flask and placed in the known weight of the evaporator flask (1000 mL). The mixture was removed with a roto-evaporator. The dried mixture was weighed and further diluted with 5 mL of dichloromethane. The mixture was placed in a glass bottle and incubated at 4 °C in the refrigerator before being analyzed with GC-FID Shimadzu QC 2010-GC-FID (Japan). The GC was equipped with three detectors: FID, TCD, and ECD with AOC-20 autosampler, and the sample were auto injected by the GC. The chromatographic column used was a Teknokroma TRB-1 (30 m × 250 μm (or 0.25 mm) × 0.25 μm) (purchased from Shimadzu, Japan). The dimethyl-polysiloxane inner coating is suitable to separate each organic compound from the mixture of organic compounds as per their retention time.

The breakdown of hydrocarbons was performed using a Shimadzu QC 2010-GC-FID. The GC-FID was programmed as column initial temperature was 70 °C and was raised till 280 °C at the heating rate of 2 °C/min for 280 °C and hold time for 3 min, or column flow rate 2 mL/min, with the complete time of 28.78 min. The injector and detector temperature was kept at 280 °C throughout the program. Around 2 µL of the sample was placed in an autosampler vial and auto-injected. After every 5 samples, a blank sample (with dichloromethane) was run to clean the column. Each sample was run in replicate.

(2) $$\mathrm{Response}\;\mathrm{Factor}\;\mathrm{of}\;\mathrm{Analyte}\;\mathrm{Standared}=\frac{\mathrm{Concertration}\;\mathrm{of}\;\mathrm{standard}}{\mathrm{response}/\mathrm{peak}\;\mathrm{areas}\;\mathrm{of}\;\mathrm{standard}}$$

The calibration curve is determined by the analysis of 3 calibration levels, i.e., 0.1, 0.25, and 0.5 mg/kg. The percentage peak area method was used for the area of the target compound peak as a proportion of the total area of all detected peaks to analyze quantity. Each peak area was calculated, and the calibration curve was calculated and plotted for concentration vs response area or peak area. Response factor was calculated through Equation (2). The calibration curves were best fitted to a linear curve. The correlation coefficients (R) were 0.9947. The quantification was performed from the mean of two calibration curves surrounding the samples.

### 2.9. Statistical Analysis

Statistical analyses were performed with Minitab and Origin8 software. Regression analysis was performed by using Minitab software, to determine the relationship between input and output variables, i.e., TPH, number of days, and treatments. Furthermore, TPHs can be related statistically with treatments and the number of days through Equation (3). Besides, the correlation between TPH and the number of days was established by Minitab software. However, the change in the rate of degradation of short, medium, and long-chain hydrocarbons and isoprenoids (with respect to time) was plotted using origin 8.0. The total petroleum hydrocarbons are the mean and standard error of three replicates. Three replicates of each treatment in different chains of hydrocarbons were analyzed by using ANOVA by using Statistix 8.1.

(3) $$\mathrm{TPH}\;=423-27.9\times \left(\mathrm{treatment}\right)-0.647\times \left(\mathrm{number}\;\mathrm{of}\;\mathrm{days}\right)$$

## 3. Results

### 3.1. Physicochemical Characteristics of Control and Petroleum Waste Soil

Table 2 shows the difference in the physicochemical and total bacterial population (CFU/g of soil) between control soil and petroleum waste contaminated soil. When 50% (*w*/*w*) of petroleum waste was extracted through Soxhlet extraction, around 404 mg/g of crude oil was extracted from the soil in which 50% (*w*/*w*) of petroleum waste was added. The soils were sandy loam and sandy clay loam in nature. Control soil was reddish yellow with 7.5Y, 7/6YR. Petroleum waste soil was 5Y-2.5/1 black on the Munsell soil color chart. Control soil was deficient in organic matter, whereas it was nearly 10.26% in petroleum waste soil. Nitrogen was 2.67 mg/kg in control and 2.45 mg/kg in petroleum waste contaminated soils. Total phosphorus was 0.85 mg/kg in control and 0.94 mg/kg in petroleum waste contaminated soils. Both soils were deficient in total nitrogen and phosphorus. The bacterial population (CFU/g) was 100-fold greater in control soils than petroleum waste soils.

### 3.2. Phylogenetic Relationship of Microbes

**Figure 1:**
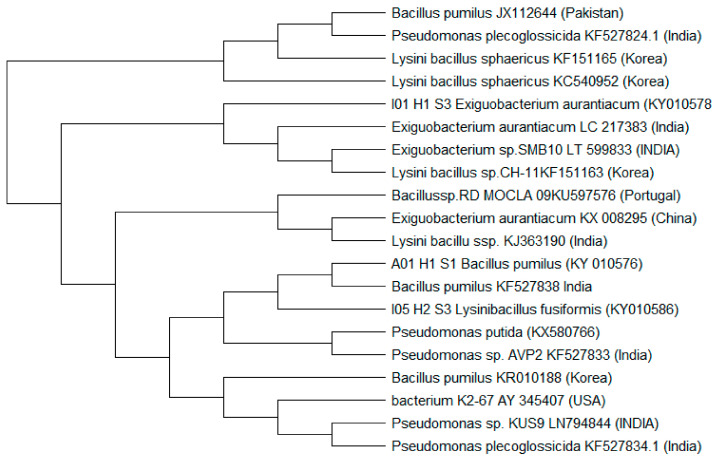
Evolutionary relationships of microbes (used for consortium preparation). The evolutionary history was inferred using the Neighbor-Joining method. The optimal tree with the sum of branch length = 68.70927977 is shown. The evolutionary distances were computed using the Maximum Composite Likelihood method and are in the units of the number of base substitutions per site. The analysis involved 20 nucleotide sequences. All ambiguous positions were removed for each sequence pair. There was a total of 900 positions in the final dataset. Evolutionary analyses were conducted in MEGA X.

Based on the phylogenetic relationship the bacterial strains (used in consortium preparation) were assigned to four groups, as shown in Figure 1. *Bacilluspumilus* was closer to *Lysinibacillus fusiformis* based on 16S rDNA fragment. Whereas, *Exiguobacterium aurantiacum* was least related to others (Figure 1).

### 3.3. Growth Curve

The AgNO_3_ growth curve was measured by inoculating consortium in AgNO_3_ at a concentration of 0.5, 1, 1.5, 2, 5, and 8 mg/kg in a separate flask and was placed over a shaking incubator. Once maximum growth was obtained, the flask was removed from the shaking incubator. It was observed that the maximum growth of bacteria was obtained at 1.5 mg/kg of AgNO_3_ after 48 h of incubation. The growth curve was monitored with a UV-VIS spectrophotometer.

### 3.4. Degradation Curve of Oil with AgNPs

The degradation curve was measured to obtain the best suitable concentration of AgNPs with the prepared consortium for the degradation of hydrocarbons. Crude oil with various concentrations (0.5, 1, 1.5, 2, 5, 8 mg/kg) of nanoparticles was shaken in a shaking incubator for seven days. The consortium was also added to it. The degradation of crude oil was observed after 24 h, 48 h, and seven days of incubation by UV-VIS Spectrophotometer (UV-2550, Shimadzu, Kyoto, Japan) at 410 nm [26].

### 3.5. Degradation of TPHs at Different Intervals of the Incubation Period

Table 1 shows the difference in the degradation of total petroleum hydrocarbons in untreated (T0) and treated soils (T1–T7) at five, 20, 60, and 240 days of incubation. It was observed at day 0 of the incubation period that the concentration of TPH in untreated soil (T0) was 404 mg/g. Thereafter, only 60 mg/g of TPHs was degraded by T0 till the end of the incubation period of 240 days. Moreover, with T1, the rate of degradation was slow initially and only 36% of TPHs were degraded after 240 days of incubation (Table 1).

T2 had no significant effect on the degradation of TPHs during the entire incubation period. In contrast, the efficiency of consortium (T3) to degrade TPHs was quite satisfactory in a short period because 50% of TPHs were degraded up to20 days of incubation. A further increase in the rate of degradation of TPHs was observed in the later stages when 85% of TPH was degraded at the 240th day of the incubation period (Table 1). Treatment 4 (T4) had no significant effect on the degradation of TPHs till 60 days of incubation. Nearly 34% of TPHs were degraded between 60–240 days of the incubation period (Table 1). T5 had no significant improvement in the degradation of TPHs when compared with T3 over a short period. Nearly 52% of TPHs were degraded over a short period, and a little amount of TPHs was present at the end of the incubation period (Table 2). Treatment 6 had no significant effect on the degradation of TPHs when compared with T3. Nonetheless, T6 increased the rate of degradation of TPHs when compared with T3 throughout the incubation period (240 days). There was a significant (*p* < 0.05) difference in the rate of degradation of TPHs between T3 and T6 over a short time (20 days) and at the end of the incubation period (60–240 days). Around 80% of TPHs was degraded by T3 alone throughout 240 days of incubation (Table 1).

Treatment 7 had a significant (*p* < 0.05) effect on the rate of degradation of TPHs when compared with To, T1, T2, T4, and T6 in a short period of incubation (20 days). Moreover, a negligible amount of TPHs was remaining at the end (240 days) of the incubation period (Table 1).

### 3.6. Degradation and Disappearance of Short, Medium, Long-Chain Hydrocarbons and Isoprenoids by T1–T7s

**Figure 2:**
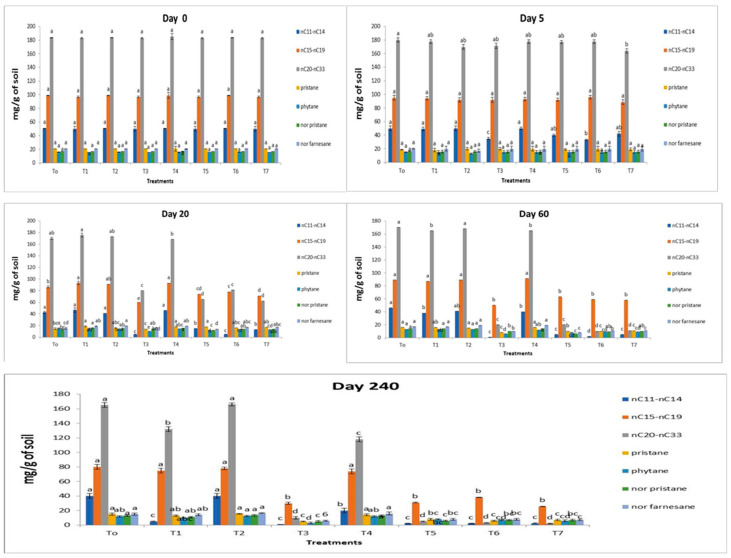
Difference in the rate of degradation of *n*-alkanes, isoprenoids, pristane, and phytane in untreated (T0) and treated (T1–T7) petroleum waste contaminated soils at different intervals (0, 5, 20, 60, and 240 days) of the incubation period. The data is the comparison among different treatments at different days and all the treatments sharing a common letter are similar otherwise differ significantly at *p* < 0.05.

The rate of degradation and disappearance of short- (*n*C_11_ to *n*C_14_), medium- (*n*C_15_ to *n*C_19_), and long-chain hydrocarbons (*n*C_20_ to *n*C_32_) and isoprenoids (nor-farnesane, nor-pristane, pristane, phytane) by treatment (T1) is shown in Figure 2. Treatment 1 had no significant effect on the rate of degradation of short, medium, or long-chain hydrocarbons over a short period. However, short- and long-chain hydrocarbons completely disappeared throughout 60–240 days of incubation. A significant number of medium-chain hydrocarbons were present at 60–240 days of incubation. Isoprenoids were degraded slowly and were present at the end of the incubation period (Figure 2).

T2 had no significant effect on the rate of degradation of all (short, medium, high, or isoprenoids) hydrocarbons till the end of the incubation period (Figure 2). Treatment T3 had a significant (*p* < 0.05) effect on the degradation of short carbon chain over a short period, whereas medium-chain hydrocarbons were degraded slowly initially (till 20 days of incubation) but completely disappeared at 240 days of incubation, and a negligible amount of TPHs was present at the end of the incubation period (240 days). However, isoprenoids (nor-farnesane, nor-pristane) disappeared at day 60 of the incubation period, whereas pristane and phytane were persistently present till the end (Figure 2).

The rate of degradation and disappearance of short, medium, and long-chain hydrocarbons and isoprenoids by T5, T6, and T7 is presented in Figure 2. These treatments followed the same trend shown by T3. There was no significant difference in the disappearance of all carbon chain hydrocarbons and isoprenoids between T5, T6, T7, and T3.

Treatment 4 shows no degradation of any carbon-chain hydrocarbons over a short period (Figure 2). There was no difference in the rate of degradation of any carbon chain hydrocarbons between T4 and T1 at 60–240 days of incubation. The rate of degradation of isoprenoids was slow and nearly 50% of these compounds were present at the end of the incubation period.

Treatments 5–7 had a significant (*p* < 0.05) effect on the degradation rate of all carbon chain hydrocarbons and isoprenoids over a short period. However, it was observed that T1 and T2 alone had no significant effect on the degradation rate of all carbon chain hydrocarbons over a short period. However, when these treatments were inoculated with consortium or T3, the rate of degradation was rapid, and nearly 70% of the TPHs were degraded over a short period. The same trend was observed with isoprenoids (Figure 2).

### 3.7. Bacterial Population (CFU/g Soil) at Different Intervals of Incubation Period

The difference in the bacterial population (CFU/g oven-dry weight of soil) between T0 and all other treatments is presented in Table 3. The results of this study show that the T0 has 2 ×10^4^ CFU/g of soil. In comparison, ‘T1’ increased the bacterial population (from 10^4^ to 10^6^) at day 60 of the incubation period. Thereafter, the bacterial population remained the same until the end of the incubation period. T3 increased the bacterial population by 100-fold at day 20 of incubation when compared with T2 and T1. Thereafter, no further increase in the bacterial population was observed till the end of the incubation period (240 days). T6 did not increase the bacterial population over an entire period of incubation. The same trend of the bacterial population was observed in T2. No difference in the bacterial population was observed between T6 and T2.

In contrast, the bacterial population had shown a significant (*p* < 0.05) increase in T5. There were 100 folds increase in the bacterial population in T7 on day 20 of incubation. However, no further growth in the bacterial population was observed until the incubation period.

### 3.8. Regression Analysis

The significance analysis of regression between treatments to days is presented in Table 4 and Table 5. The regression analysis results show that T3, T5, T6, and T7 led to a significant difference (*p* < 0.05) compared toT1, T2, and T4 of TPHs at days 20, 60, and 240 of the incubation periods. Individual *p*-values of both factors, i.e., treatments and number of days, are 0.00011 and 0.00019, respectively. Both these values are significantly less than 0.05. This further implies that both factors (treatments and number of days) tremendously affect the TPHs values. Furthermore, TPHs can be related statistically with treatments and the number of days through Equation (4).

(4) $$\mathrm{TPH}\;=423-27.9\times \left(\mathrm{treatment}\right)-0.647\times \left(\mathrm{number}\;\mathrm{of}\;\mathrm{days}\right)$$

### 3.9. Difference in the Rate of Degradation of TPHs w.r.t Treatment &Time

**Figure 3:**
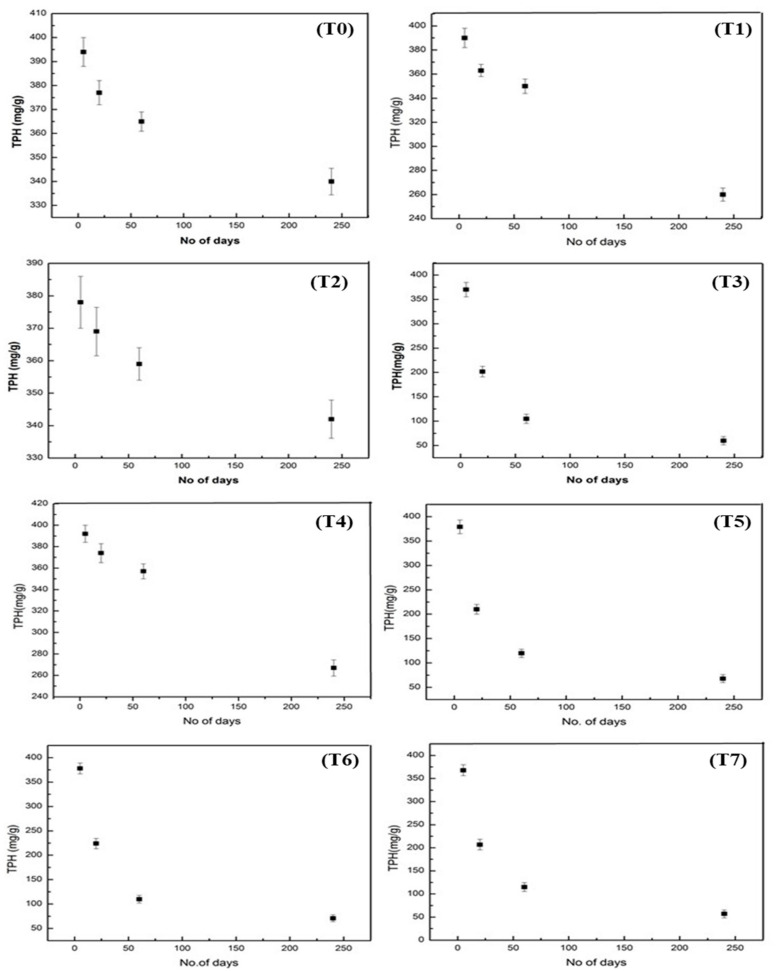
The difference in the degradation of TPH with respect to time (No. of days) in treatments (T1–T7) and (T0). The values are the mean and standard error ± of three replicates.

The difference in the rate of degradation of TPHs in the treated (T1–T7) and untreated (T0) samples at various days of incubation is presented in Figure 3. The result of this study shows that the degradation of TPHs was significantly (*p* < 0.05) increased in treatments with consortiums such as T3, T5, T6, and T7 in relation to days of incubation. For treatments T0, T1, and T2, slow degradation of TPHs was recorded, whereas in treatments T3, T5, T6, and T7, rapid degradation of TPHs were observed. The results show that T0 and T2 degraded a negligible amount of TPHs until the incubation period (240 days). However, T1 and T4 led to comparatively better degradation than T0 and T2. In contrast, T3 T5, T6, and T7 significantly (*p* < 0.05) accelerate the degradation of TPHs over a short period.

## 4. Discussion

A limitation in bioremediation revealed the contention that the complete remediation of petroleum waste contaminated soils requires some additional accelerators with nontoxic characteristics and a large surface area [27,28]. AgNPs, because of their environmental feasibility, ecologically sound behavior, and availability of large surface area for microbes, were known to remediate industrial wastewater [29,30,31,32]. However, studies related to the use of AgNPs to remediate petroleum waste contaminated soils provide inconsistent findings. The present study shows that AgNPs application alone at 1.5 mg/kg concentration to petroleum waste contaminated soils does not positively or negatively affect the bacterial population and cannot accelerate the degradation of TPHs. Other metallic nanoparticles [33] are considered toxic to microbes when mixed with soil [34,35].

It was observed in this study that fertilizer addition increased the rate of degradation to 36% of TPHs at the end of the incubation period (Table 2). Several previous studies have suggested that fertilizer addition to petroleum waste enhances the rate of degradation of TPHs in the later stages of the incubation [29,30]. Enough source nitrogen was available for microbes to degrade TPHs at the end of the incubation period. This may also reveal the contention that indigenous microbes become active in the presence of available nutrient sources to degrade TPH.

The present study results show that AgNPs alone did not accelerate the degradation of TPHs. Hence, no noticeable increase in the bacterial population has been noticed in such soils (Table 3). The observation suggests that AgNPs may not effectively enhance bacterial growth to degrade the total petroleum hydrocarbons in the petroleum waste-contaminated soils. The result of this study is in conformance with the conclusion drawn by Beddow and colleagues [36], who found that AgNPs do not appear to affect the ability of microbes to biodegrade oil. They also reported that total bacterial counts were lower with AgNPs compared to the soil without AgNPs. Furthermore, their study concluded that AgNPs inhibit total bacterial counts in soils contaminated by petroleum hydrocarbons-.

This study also shows that fertilizer addition alone causes the disappearance of any of the short-chain hydrocarbons later in the incubation period (60–240 days). Nitrogen and phosphorus are essential nutrients required for the microbial assimilation of hydrocarbons [37,38]. Nitrogen and phosphorus requirements in petroleum waste contaminated soils depend on the contamination’s extent and type. Moreover, it was evident from the previous literature that the optimum ratio of nitrogen to phosphorus is 10:1 for petroleum waste contaminated soils [39,40]. Nitrogen and phosphorus are deficient in petroleum waste contaminated soils [41]. This deficiency can be overcome by adding nutrient supplements in the form of fertilizer, such as ammonium nitrate, urea, di-ammonium phosphate, or ammonium sulfate, to petroleum-contaminated soils. This may accelerate the degradation of total petroleum hydrocarbons [42]. However, the nitrogen and phosphorus concentration added to the soil to achieve promising results is debatable. Nitrogen and phosphorus at a 10:1 ratio are considered optimum to enhance the degradation of hydrocarbons when added to the soil impregnated with petroleum [43,44,45]. This study shows that nitrogen to phosphorus at 10:1 ratio (T1) was enough to degrade total petroleum hydrocarbons (TPHs) at day 60–240 of the incubation period (Figure 2). With the addition of fertilizer to the petroleum waste contaminated soils, nearly 36% of TPHs was degraded at the 60th day of incubation period. The above-mentioned results are linked with the increase in the bacterial population at days 60–240 of the incubation period. This is in consistence with the findings of [46]. They reported that the effect of fertilizer (NPK) to degrade diesel hydrocarbons in the soil was evident at the 21st–28th day of incubation. They also concluded that this is because of the increased microbial biomass in such soils. Wu and colleagues [29] reported that nitrogen ((NH_4_)_2_SO_4_) and phosphorus (KH_2_PO_4_) addition to crude oil contaminated soils accelerates the degradation of total petroleum hydrocarbons throughout 70 days. They also found that TPH was reduced from 46,600 mg/kg of soil to 18,400 mg/kg of soil by treating with fertilizer. Mohammadi-Sichani [47] also reported that fertilizer addition degraded 64.7% of total petroleum hydrocarbons in comparison to soils without fertilizer and compost, whereas Siles and Margesin [48] reported that when the addition of NPK at a C: N ratio of 20:1 was added to the crude oil-contaminated soil, it mineralized around 74.7% of TPHs. This is linked with abundance in the bacterial population in the treated soils.

As discussed above, adding nitrogen and phosphorus at a ratio of 10:1 was enough to degrade petroleum waste-contaminated soils at 60–240 days of incubation. This study showed that consortium with fertilizer addition to petroleum waste contaminated soils reduces the lag phase from 60 to 20 days. These findings are consistent with the increase in the colony-forming unit in the contaminated soils. Fertilizer alone degraded 36% of TPHs at day 60, whereas consortium addition to such soil increased the TPHs degradation to 52% at day 20. These findings are consistent with Mishra and colleagues’ findings [48]. They reported that consortium addition with fertilizer assimilates TPHs rapidly compared to fertilizer alone. Their study indicated that almost 92% of TPHs were removed from the soil at 360 days of incubation. Similar results were also reported by Fitri and colleagues [49], Shah and others [50], Hamza and colleagues [51], Ishrat [52], Mittal and coworkers [53], and Singh and others [54]. It is already known that petroleum waste contaminated soil is a hub of thousands of hydrocarbon-degrading genera. The bioremediation technique is practiced worldwide to enhance the degradation rate of petroleum waste by using these naturally occurring petroleum hydrocarbon degraders [55,56,57]. *Pseudomonas* and *Bacillus* predominate petroleum waste contaminated soils [58]. Their application as a bio-degrader has been formulated successfully across the globe [59,60,61]. However, their efficiency in combination with AgNPs to remediate petroleum waste-contaminated soil is not yet reported. Our study also validates that *Pseudomonas putida* (acc no. KX580766), *Bacillus pumilus* (Acc KY010576), *Exiguobacteriaum aurantiacum* (Acc KY010578), and *Lysinibacillus fusiformis* (Acc KY010586) are efficient in the degradation of total petroleum hydrocarbons (TPHs). The consortium prepared from these genera is effective and degraded TPHs in a short incubation time. This is in agreement with the findings of Piñón-Castillo and colleagues [62], Goetz and coworkers [63], M’rassi and colleagues [64], and Ali and others [65], who reported that single strain *Acinetobacter* sp. A3 is capable of degrading 70% of TPH from crude oil-contaminated soils. However, Ali and colleagues [65] inoculated the hydrocarbon contaminated soil with the consortium (*Pseudomonas* and *Marinobacter*) and degraded the total petroleum hydrocarbons rapidly. Similar results of degradation (using different consortiums) were also reported by [21,66,67,68,69].

Kumar and colleagues [67] found that *Pseudomonas aeruginosa* M and NM consortia and *Bacillus subtilis* effectively decontaminating the petroleum contaminated soils for 120 days. As discussed above, a consortium can accelerate the degradation rate of petroleum hydrocarbons from the soil. The study, as mentioned earlier, explained the use of *Pseudomonas putida* (acc no. KX580766) and *Bacillus pumilus* (Acc KY010576) as an inoculum to remediate petroleum waste contaminated soils. However, a mixed consortium of the above-mentioned strains with *Exiguobacteriaum aurantiacum*(Acc KY010578) and *Lysinibacillus fusiformis* (Acc KY010586) is reported in this study for the first time. Promising results were obtained, confirming the use of consortium at a commercial scale.

The efficiency of AgNPs in combination with the consortium of bacterial strains to degrade TPH and enhance the bacterial population was examined in this study. Our results depicted that AgNPs had no harmful effect on the consortium. The consortium in combination with AgNPs removed approximately 50% of TPH at day 20 of incubation (Figure 2). This suggests that the consortium prepared in this study was suitable to remediate TPH in the petroleum waste contaminated soils. This is in correlation with a 100-fold increase in colony-forming units (Table 3). The presence of *Pseudomonas* and *Bacillus* strains in our consortium show that such bacterial strains can survive under an AgNPs stressed environment. The above-mentioned strains can contribute efficiently to degrade TPH and increase the bacterial population.

Limited information is available regarding AgNPs as bio-degraders. [70] found that AgNPs as adsorbents can remove 85% hydrocarbons (Phenanthrene > Pyrene > Anthracene). Some of the previous studies were focused on understanding the role of metallic nanoparticles to degrade crude oil in the laboratory. Kumar and colleagues [67] did not find a positive effect of iron nanoparticles in the remediation of crude oil.

The addition of AgNPs in combination with the consortium in petroleum waste contaminated soils may not accelerate the degradation rate of total petroleum hydrocarbons. However, this study also shows that inoculated hydrocarbon degraders remained ineffective with the addition of AgNPs to petroleum waste-contaminated soils. This suggests that AgNPs’ antimicrobial activity was ineffective at a lower concentration (1.5 mg/kg). AgNPs may not be present in their original form after a certain incubation period. In addition, the presence of organic compounds may alter the original form of added AgNPs in the petroleum waste. Hence, AgNPs become inactive to elevate the mineralization of total petroleum hydrocarbons in the presence of hydrocarbon degraders [28,71].

The petroleum waste used in our study was composed of *n*-alkanes and isoprenoids, where 80% were *n*-alkanes (*n*C_11_ to *n*C_33_) and 10% were isoprenoids. When consortium was added to petroleum waste contaminated soils, the degradation rate of short and long-chain hydrocarbons was accelerated. Approximately 30% short-chain and 20% long-chain hydrocarbons were degraded on day 20. Besides, only 10% of medium-chain hydrocarbons were degraded at day 20 of incubation. After that, *n*-alkanes almost disappeared on day 240 of incubation. Isoprenoids also completely disappeared. The result of this study is not consistent with the findings of Gazali and coworkers [60]. They reported that a consortium prepared from *Bacillus* and *Pseudomonas* sp. was effective to degrade medium and long carbon chain *n*-alkanes in the soil impregnated with engine oil over 30 days. Whereas, Maletic and colleagues [63] reported that *n*-alkanes present in crude oil-contaminated soils with middle chain hydrocarbons were degraded rapidly in comparison to long-chain hydrocarbons. They concluded that the delay in the degradation of long carbon chain hydrocarbons is due to their hydrophobic nature, making it difficult for microbes to degrade rapidly. The degradation of short chain hydrocarbons with consortium was also reported by Bento and colleague [72] and Naeem and coworkers [73]. They reported that a consortium composed of *Bacillus cereus*, *Bacillus sphaericus*, *Bacillus fusiformis*, *Bacillus pumilus*, *Acinetobacter junii*, and *Pseudomonas* sp. can accelerate the degradation of short-chain hydrocarbons, whereas long-chain hydrocarbons were degraded slowly at the end of the incubation period (240 days).

Regression analysis also validates those ‘treatments’ (T1–T7) and the ‘number of days’ significantly affects the TPHs (Table 4 and Table 5). Individual *p*-values of both factors, i.e., treatments and number of days, are 0.00011 and 0.00019, respectively. Both these values are significantly less than 0.05. This further implies that both factors and the number of days tremendously affect the TPHs values.

## 5. Conclusions

In conclusion, amendment with a consortium of hydrocarbon-degrading bacteria degraded 70% more TPHs consortium than all other treatments over a short period. This degradation is mediated by microbes more likely because there was enough carbon source available for microbes to perform their activity. Fertilizer alone or in combination with AgNPs and consortium slows the rate of degradation of TPHs over a short period, but later on, enhances the rate of degradation of TPHs, and a negligible amount remained at the end of the incubation period. However, silver nitrate nanoparticles (AgNPs) alone had no significant effect on the rate of degradation of TPHs. Therefore, it is recommended to investigate the degradation potential of carbon-based nanoparticles in the future for the degradation of petroleum hydrocarbons. This study also revealed that the efficiency of AgNPs under viscous unrefined oil is negligible. This may be because the highly viscous oil is more resistant to degradation than lighter oil. Therefore, it is recommended to apply metal nanoparticles to lightweight refined oil than highly viscous petroleum waste in the future.

## Figures and Tables

**Table 1:** The difference in the degradation of total petroleum hydrocarbons in petroleum waste (T0) and other treated soils (T1–T7) at 5, 20, 60, and 240 days of incubation.

Treatment		TPH (mg.g^−1^ of Soil)			
		Days of Incubation			
		5	20	60	240
T0	Petroleum waste only	394 a*	377 a	365 a	340 a
T1	Petroleum waste +Fertilizer	390 a	363 bc	350 bc	260 b
T2	Petroleum waste + nanoparticle	378 b	369 b	359 b	342 a
T3	Petroleum waste + consortium	370 bc	202 ef	105 e	60 cd
T4	Petroleum waste + fertilizer + nano particle	392 a	374 a	357 b	267 b
T5	Petroleum waste + fertilizer + consortium	379 b	210 e	120 e	68 c
T6	Petroleum waste + nano particle +consortium	378 b	224 d	110 de	71 c
T7	Petroleum waste + fertilizer + consortium + nano particle	368 bc	207 e	115 de	57 d

* The data is the mean of three replicates. The data is the comparison among different treatments at different days and all the treatments sharing a common letter are similar otherwise differ significantly at *p* < 0.05.

**Table 2:** Control and petroleum waste physicochemical and total bacterial population (CFUs/g of soil).

Soil Types	Control	Petroleum Waste (Added 50% to Soil)
Crude oil extracted (mg per g)	Nil	404
Emulsion of water and waste (mg per g)	Nil	96
pH	8.98	8.38
EC (dS cm^−1^)	57.1	63.1
Texture	Sandy clay loam	Sandy loam
Color (Hue-value/chroma)	7.5YR-7/6-Reddish yellow	5Y-2.5/1 black
Nitrate-Nitrogen (mg/kg)	2.671	2.45
Total Phosphorus (mg/kg)	0.85	0.94
Organic matter (%)	0.969	10.268
Total bacterial population (CFU/g of soil)	2 × 10^6^	285 × 10^4^

**Table 3:** The difference in the colony forming units (CFUs/g of over dry soil) between petroleum waste (T0) and other treated petroleum waste soils (T1–T7) at 5, 20, 60, and 240 days of incubation.

Treatments		CFUs/g of Oven Dry Soil			
		Days of Incubation			
		5	20	60	240
T0	Petroleum waste only	2.3 × 10^4^ a*	2.4 × 10^4^ b	2.9 × 10^4^ c	3.1 × 10^4^ b
T1	Petroleum waste + Fertilizer	2.3 × 10^4^ a	2.4 × 10^4^ b	2.9 × 10^6^ d	31 × 10^6^ c
T2	Petroleum waste + nano particle	2.4 × 10^4^ a	2.4 × 10^4^ b	2.8 × 10^4^ c	3.0 × 10^4^ b
T3	Petroleum waste + consortium	2.3 × 10^4^ a	2.4 × 10^6^ c	2.9 × 10^6^ d	31 × 10^6^ c
T4	Petroleum waste + fertilizer + nano particle	2.3 × 10^4^ a	2.4 × 10^6^ c	2.9 × 10^6^ d	3.1 × 10^6^ c
T5	Petroleum waste + fertilizer + consortium	2.3 × 10^4^ a	2.6 × 10^6^ c	3.2 × 10^6^ b	3.3 × 10^6^ c
T6	Petroleum waste + nano particle + consortium	2.3 × 10^4^ a	2.4 × 10^6^ c	2.9 × 10^6^ d	3.1 × 10^6^ c
T7	Petroleum waste + fertilizer + consortium + nano particle	2.3 × 10^3^ b	3.4 × 10^6^ a	3.44 × 10^6^ a	3.60 × 10^6^ a

* The data is the mean of three replicates. The data is the comparison among different treatments at different days and all the treatments sharing a common letter are similar otherwise differ significantly at *p* < 0.05.

**Table 4:** The statistical analysis between treatments and number of days using Minitab software-based.

Predictor	Coefficient	SE Coefficient	*t*-Value	*p*-Value
Constant	422.65	30.84	13.71	<0.000001
Treatments	−27.866	6.661	−4.18	0.000111
No of days	−0.6472	0.1626	−3.98	0.000193

**Table 5:** The regression analysis between treatments and number of days using Minitab software.

Analysis of Variance					
**Source**	**DF**	**SS**	**MS**	**F-Value**	***p*-Value**
Regression	2	248,481	124,240	16.67	0.000015
Residual Error	29	216,143	7453		
Total	31	464,624			

## Data Availability

The datasets used and/or analyzed during the current study are available in the tables and figure or you can get from the corresponding author on request.
